# Late diagnosis and effective everolimus treatment in a familial case of tuberous sclerosis complex: a case report

**DOI:** 10.3389/fgene.2026.1772908

**Published:** 2026-02-23

**Authors:** Fang Dai, Yulian Duan, Bao-an Di, Qiang Feng, Jing Cui, Tao Lv

**Affiliations:** 1 College of Chemistry and Material Engineering, Qujing Normal University, Qujing, Yunnan, China; 2 Neurology Department, 920th Hospital of People’s Liberation Army Joint Logistic Support Force, Kunming, China; 3 Pathology Department, 920th Hospital of People’s Liberation Army Joint Logistic Support Force, Kunming, China; 4 College of Biological Resource and Food Engineering, Qujing Normal University, Qujing, Yunnan, China

**Keywords:** angiofibromas, autosomal dominant inheritance, everolimus (6442177), TSC gene, tuberous sclerosis

## Abstract

**Background:**

Tuberous sclerosis complex (TSC) is an autosomal dominant neurocutaneous disorder. Despite established genetic causes, missed or late diagnosis remains common in familial cases. This study reports a familial case of TSC to highlight the diagnostic challenges and evaluate the clinical efficacy of everolimus in managing cutaneous and neurologic symptoms.

**Case presentation:**

The patient presented with refractory seizures, facial angiofibromas, and intellectual disability. Sequencing analysis revealed a mutation in the *TSC2* gene in both the patient and the mother: c.848 + 281 (IVS9) C > T. No mutation at this site was detected in the father. Following the diagnosis, the patient received treatment with everolimus. A significant reduction in seizure frequency and improvement in facial angiofibromas were observed during the follow-up period.

**Conclusion:**

A heterozygous splicing mutation in the TSC2 gene was identified, confirming the diagnosis of familial TSC. This case underscores the importance of genetic testing in suspected cases to prevent late diagnosis. Furthermore, our findings support the effectiveness of everolimus as a therapeutic option for alleviating TSC-associated neurological and cutaneous manifestations.

## Introduction

Tuberous sclerosis complex (TSC) is an autosomal dominant, multisystem neurocutaneous disorder, also known as Bourneville disease, that can affect virtually every organ in the body. TSC is a rare disease, with an estimated incidence of approximately 1 in 10,000 in adults and 1 in 6,000 in newborns, and shows no significant gender differences (Northrup et al., 2021). Approximately 70% of TSC cases occur sporadically, while the remaining 30% have a positive family history (Henske et al., 2016). However, the clinical presentation can be highly variable and age-dependent, which often leads to missed or late diagnosis, even in familial cases. Typical clinical manifestations include facial angiofibromas, seizures, and intellectual impairment. The genetic heterogeneity of TSC has been confirmed by genetic linkage analysis, which have demonstrated that disease is primarily associated with mutations in two genes, *TSC1* and *TSC2*, encoding hamartin and tuberin, respectively. Hamartin and tuberin readily form heterodimers within cells and regulate critical cellular processes, including cell proliferation, adhesion, and endocytosis, through the mammalian target of rapamycin (mTOR) signaling pathway (Au et al., 2007; Ehninger et al., 2010). Consequently, mTOR inhibitors, such as everolimus, have emerged as targeted therapies that can effectively alleviate TSC-associated symptoms. In this study, we report a familial TSC case from a Han Chinese family in Yunnan province, China. Beyond identifying the genetic mutation, we aim to highlight the challenge of late diagnosis in this genodermatosis and report the favorable response of both cutaneous and neurologic symptoms to everolimus treatment.

## Clinical data

**FIGURE 1:**
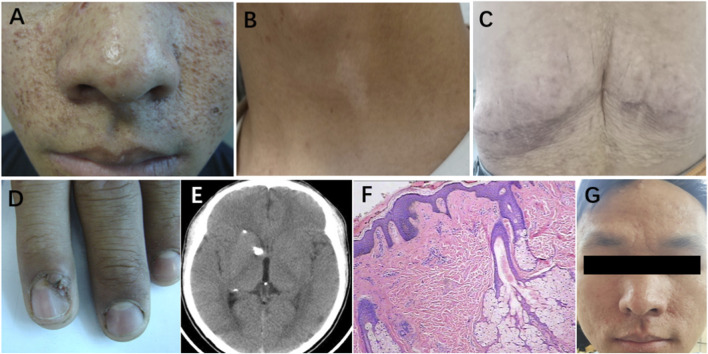
Clinical manifestations of patients with tuberous sclerosis. **(A)** Facial angiofibromas. **(B)** Hypomelanotic macules. **(C)** Shagreen patch. **(D)** Ungual fibromas. **(E)** Subependymal calcified nodules on CT. **(F)** Facial angiofibromas (H&E staining). **(G)** Improvement of facial angiofibromas.

The patient was 15-year-old unmarried and childless individual who was admitted with dizziness accompanied by a heavy sensation in the head upon waking in the morning for 7 days, along with generalized weakness. The symptoms were not significantly relieved by rest. There was no history of nausea, vomiting, blurred vision, headache, fever, slurred speech, loss of consciousness, limb movement disorders, or mental or behavioral abnormalities. Past medical history: The patient underwent surgery for “lumbar tumor resection” at the age of four (details unknown). Six months later, the patient sought repeated treatment in the dermatology department (details unknown) due to the appearance of multiple facial papules. Since childhood, the patient had exhibited slow responses and poor academic performance but had no history of seizures. Family history: The patient’s mother has facial papules but no history of seizures or drug allergies. Physical examination: Multiple small papules were observed on the facial skin, with hair growth, and new growths were noted on the fingertips. Neurological examination: The patient was alert and oriented, with appropriate speech and good cooperative during the examination. Orientation, memory, and calculation abilities were intact. Visual acuity was normal. Bilateral pupils were equal and round, approximately 3 mm in diameter, with sensitive pupillary light reflexes. Extraocular movements were intact without nystagmus or diplopia. No enophthalmos or exophthalmos was observed, and corneal reflexes were normal. Oral and cranial nerve examination: The mouth opened symmetrically without deviation. The tongue protruded in the midline, and no tremor or hypertrophy of the tongue muscles was observed. Motor and coordination examination: Muscle strength in all limbs was graded as V, with normal muscle tone. Finger-to-nose, heel-to-shin, and rapid alternating movement tests were performed smoothly. Romberg’s sign was negative. Deep tendon reflexes were symmetrical in all limbs. No pathological reflexes were detected in the lower limbs, and meningeal irritation signs were negative. Radiological examination: Brain computed tomography (CT) revealed the following findings (Figure 1E: Multiple nodular hyperdense lesions beneath the bilateral lateral ventricles and along the ependyma, suggestive of calcified nodules. Edematous lesions in both cerebral hemispheres. Demyelinating changes in the periventricular white matter adjacent to the lateral ventricles. Second admission (2022): At the age of 26, the patient was readmitted with a six-month history of episodic loss of consciousness accompanied by limb convulsions. The patient reported an initial episode of loss of consciousness in mid-March 2022, followed by spontaneous recovery within a few minutes. Since April 2022, recurrent episodes of loss of consciousness and limb convulsions occurred while resting in bed, without obvious triggers. During these episodes, the patient exhibited upward rolling of the eyes and generalized body stiffening, consistent with tonic–clonic seizures lasting approximately 20 s, followed by gradual recovery of consciousness within about 2 min.

## Case description

**FIGURE 2:**
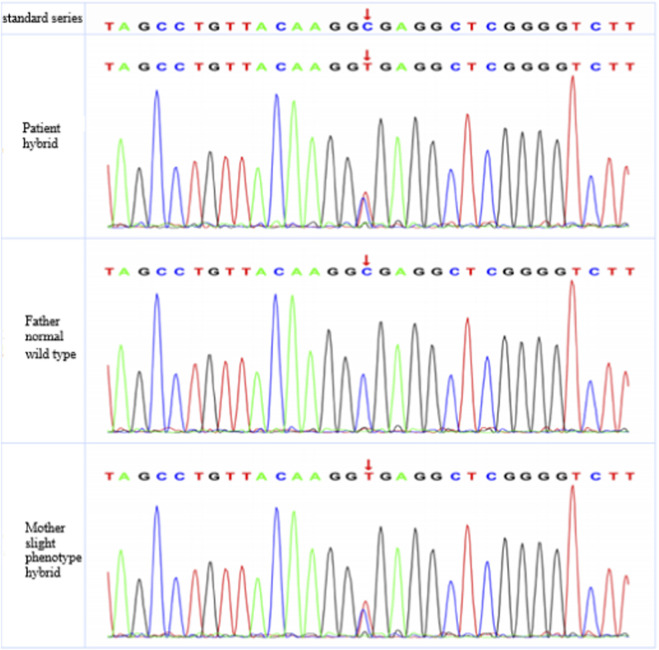
Exon sequencing results of *TSC2* gene. DNA was extracted from peripheral blood samples of the patient and his parents. Specific primers were used to amplify the *TSC2* gene, followed by Sanger sequencing on ABI 3730 sequencer. Sequence alignment was conducted using sequence analysis software.

The patient primarily presented with facial angiofibromas (Figure 1A), hypomelanotic macules (Figure 1B), a shagreen patch (Figure 1C), ungual fibromas (Figure 1D), and subependymal calcified nodules (Figure 1E). According to the updated 2021 international Tuberous Sclerosis Complex Consensus diagnostic criteria, the patient was diagnosed with TSC (Northrup et al., 2021). The facial lesions appears as rough, nodular eruptions. Microscopic examination revealed lobulated lesions connected to the epidermis. The lobules consisted of well-differentiated, vacuolated sebaceous gland cells. In the dermis, proliferation of fibrous tissue and blood vessels was observed. Routine histopathological examination using hematoxylin and eosin (H&E) staining confirmed the diagnosis of facial angiofibromas (Figure 1F). DNA sequencing and sequence alignment identified a mutation in the *TSC2* gene in both the patient and his mother: c.848 + 281(IVS9) C>T, indicating a cytosine-to-thymine substitution at nucleotide position 848 in intron 9 of the *TSC2* gene. This mutation was not detected in the patient’s father (Figure 2). The patient was heterozygote for this mutation, which is consistent with the pathogenic mechanism of an autosomal dominant inherited disorder. Follow-up data revealed that from 2011 to 2022, the patient was able to perform activities of daily living independently, and the facial rash showed improvement (Figure 1G). However, seizures occurred in 2021.

## Discussion

TSC is a multisystem disorder inherited in an autosomal dominant manner and is closely associated with mutations in the *TSC1* or *TSC2* genes. It is characterized by developmental abnormalities of cells and tissues affecting multiple organs. The *TSC1* and *TSC2* genes are located on different chromosomes and share no significant sequence homology. Mutations affecting functional regions of *TSC1* or *TSC2* often result in more severe clinical manifestations, including an increased risk of seizures, intellectual disability, facial angiofibromas, and renal angiomyolipomas (Wilbur et al., 2017). Previous studies have shown that, compared with *TSC1* mutations, patients harboring *TSC2* mutations tend to have an earlier onset of seizures, a higher prevalence of cognitive impairment, a greater number of cortical tubers, and a larger proportion of brain volume occupied by tubers (Van Eeghen et al., 2012). Early detection of cutaneous lesions is therefore crucial for the diagnosis of TSC (Nunes et al., 2024). In the present case, the clinical manifestations were distinctive, with early and characteristic cutaneous features. However, the patient was not diagnosed until the age of 15. At that time, in addition to cutaneous manifestations, neurological symptoms such as epilepsy had already developed. This delayed diagnosis highlights the need to improve awareness, education, and early recognition of TSC, as timely diagnosis may improve quality of life and reduce long-term sequelae.

Mutations in the *TSC1* and *TSC2* gene can be classified into five major types: missense mutations, nonsense mutations, frameshift mutations, splice site mutations, and large deletions. Among these, missense, splice site, and frameshift mutations are the most common (Dabora et al., 2001). To date, 836 mutations have been identified in the *TSC1* gene and *2345* mutations in the *TSC2* gene, indicating a significantly higher mutation frequency in *TSC2* (Northrup et al., 2013). In the present case, both the patient and his mother carried a mutation in the *TSC2* gene, involving a cytosine-to-thymine substitution at nucleotide position c.848 + 281 in intron 9, resulting in a splice site mutation. Van Eeghen et al. reported sporadic cases with mutations at this site, in which patients harbored *de novo TSC2* mutations without affected family members (Tyburczy et al., 2015). In contrast, the present study identified the same mutation in both the patient and his mother, suggesting familial inheritance of the *TSC2* c.848 + 281 (IVS9) C>T mutation. Genetic analysis demonstrated that the patient was heterozygous for this variant. Segregation of genotype and phenotype was observed within the family, consistent with the pathogenic mechanism of an autosomal dominant inherited disorder.

According to the 2019 Sequencing Variant Interpretation Guidelines established by the American College of Medical Genetics and Genomics and the Association for Molecular Pathology (ACMG guidelines), the identified variant in this case was classified as pathogenic (Richards et al., 2015). The patient initially presented at the age of 15 with facial angiofibromas, ungual fibromas, and mild intellectual impairment, with cranial CT revealed subependymal calcified nodules. Histopathological examination further confirmed the diagnosis of facial angiofibromas. By the age of 26, in addition to persistent cutaneous manifestations, the patient developed epileptic seizures, indicating multisystem involvement, particularly of the brain and skin. Although both the patient and his mother carried the same pathogenic variant, the mother exhibited only mild facial lesions. This discrepancy suggests phenotypic heterogeneity and incomplete penetrance, which may be influenced by factors such as sex, age of onset, secondary environmental insults, and other unidentified genetic modifiers (Crino et al., 2010; Hall et al., 2021).

Dysregulation of the mTOR signaling pathway is considered the molecular basis of TSC pathogenesis, with excessive mTOR activation representing a key pathogenic mechanism associated with *TSC1* and *TSC2* mutations (Uysal and Şahin, 2020). As a critical therapeutic target in *TSC*, mTOR inhibitors-particularly mTORC1 inhibitors-have been increasingly applied in clinical practice, with favorable outcomes (Franz et al., 2013). Everolimus has been demonstrated to be safe, effective, and well tolerated in patients with TSC. Oral mTOR inhibitors not only reduce hamartoma burden but also exhibit significant antiepileptic effects (Krueger et al., 2013). Several studies have reported that approximately 60% of the patients experienced improvement in their skin lesions after 6 months of everolimus treatment. In the present case, the patient was followed for 11 years and received oral everolimus at a dose of 5 mg once daily for 6 months. Sodium valproate was administered for seizure control. Following systematic treatment, both cutaneous lesions and epileptic symptoms improved, and the patient was able to live independently.

## Conclusion

We reported a familial case of tuberous sclerosis complex caused by a splice site mutation in the TSC2 gene. This case highlights two critical clinical implications. First, TSC can present with highly variable phenotypes even within the same family; therefore, clinicians must be vigilant about cutaneous signs to prevent missed or late diagnoses, particularly in patients with mild family history. Second, therapeutic intervention with the mTOR inhibitor everolimus demonstrated significant efficacy in alleviating both cutaneous and neurological symptoms. Our findings support the use of genetic testing for early definitive diagnosis and highlight the potential of precision medicine in managing this multisystem disorder.

## Data Availability

The original contributions presented in the study are included in the article/supplementary material, further inquiries can be directed to the corresponding author.
